# Spatial Variation of Soil Organic Carbon and Total Nitrogen in the Coastal Area of Mid-Eastern China

**DOI:** 10.3390/ijerph14070780

**Published:** 2017-07-14

**Authors:** Yan Xu, Lijie Pu, Qilin Liao, Ming Zhu, Xue Yu, Tianying Mao, Chenxing Xu

**Affiliations:** 1 School of Geographic and Oceanographic Sciences, Nanjing University, Nanjing 210023, China; xuyanxiaozz@126.com (Y.X.); zhumingnju@126.com (M.Z.); yx1990510@126.com (X.Y.); mty0423@163.com (T.M.); mg1527018@smail.nju.edu.cn (C.X.); 2 Key Laboratory of the Coastal Zone Exploitation and Protection, Ministry of Land and Resources, Nanjing 210024, China; 3 School of Environmental Science and Engineering, Suzhou University of Science and Technology, Suzhou 215009, China; 4 Geological Survey of Jiangsu Province, Nanjing 210028, China; 13951668257@163.com

**Keywords:** ecosystem services, coastal reclamation, carbon sequestration, prograding coast

## Abstract

Soils play an important role in sequestrating atmospheric CO_2_. Coastal tidal flats have been intensively reclaimed for food security and living spaces worldwide. We aimed to identify the changes of soil organic carbon (SOC) and total nitrogen (TN) following coastal reclamation and their spatial variation in the coastal area of mid-Eastern China to provide information for coastal cropland management. We measured SOC and TN of 463 soil samples in the coastal plain of mid-Eastern China. The results showed that SOC and TN increased highly from the uncultivated coastal tidal flat (2.49 g·kg^−1^ and 0.21 g·kg^−1^, respectively) to the cropland (10.73 g·kg^−1^ and 1.3 g·kg^−1^, respectively). After long-term cultivation, SOC and TN in the old farmland (12.98 g·kg^−1^ and 1.49 g·kg^−1^, respectively) were greater than those in the young farmland (5.76 g·kg^−1^ and 0.86 g·kg^−1^, respectively). The density of SOC in the uncultivated coastal tidal flat, young farmland, and old farmland were 0.68 kg·C·m^−2^, 1.52 kg·C·m^−2^, and 3.31 kg·C·m^−2^, respectively. The density of TN in the uncultivated coastal tidal flat, young farmland and old farmland were 0.05 kg·N·m^−2^, 0.23 kg·N·m^−2^, and 0.38 kg·N·m^−2^, respectively. The C/N (11.17) in the uncultivated coastal tidal flat was highest comparing to that in the young and old farmland due to lower nitrogen. The C/N increased from 6.78 to 8.71 following cultivation. Reclaimed coastal tidal flats had high carbon and nitrogen sequestration potential that not only mitigated the threat of global warming, but also improved soil fertility for crop production. Coastal management of cropland should consider the spatial distribution of SOC and TN to improve ecosystem services of coastal soils.

## 1. Introduction

Soils store 1462–1576 Pg of carbon (C) in the upper 100 cm globally [1,2,3] and store 684–724 Pg of carbon in the upper 30 cm [2]; these amounts are greater than those stored in both the atmosphere and vegetation [4]. Emissions of N_2_O from soils represent 33–39% of all global N_2_O sources [5]. Therefore, soils play an important role in global changes, which have direct impacts on human health. Soil carbon and nitrogen are greatly affected by land use changes [6,7,8], exotic plant invasion [9,10], and crop systems [11] in forest, coastal, and artificial ecosystems [10,12,13,14,15,16]. Coastal tidal flats are small and scattered, although they release negligible trace gases and store a great amount of carbon [17]. Many countries, such as the Netherlands, South Korea, Singapore, Japan, Spain, and China, have intensively reclaimed coastal tidal flats for living space and food security [18,19,20]. Soil organic carbon (SOC) and total nitrogen (TN) of coastal tidal flats changed greatly due to reclamation. Therefore, understanding the changes in SOC and TN following reclamation and spatial variation in coastal areas is required for coastal cropland management for ecosystem services, such as carbon and nitrogen sequestration and soil fertility.

Studies of temporal changes have utilized long-term observations, although only a few studies have covered several decades. Therefore, temporal trends can be inferred by studying sites with different ages, called space for time substitution (SFT). Space for time substitution is an important tool for analysing soil development across multiple time scales [21] and has been used in different studies [22,23] to analyse the temporal changes of SOC. However, changes in the SOC and TN in the coastal area over thousands of years were still unclear.

From the 1950s to the 1990s, over 30% of the coastal tidal flats in China were reclaimed for agriculture, salt production, and mariculture [20,24], and coastal reclamation will continue in the future [25]. In Eastern China, many coasts are prograding to the sea [26]. Due to a high coast accretion rate, the Chinese government plans to develop more cropland in the Jiangsu coastal plain from 2009 to 2020 to support national food security. Therefore, we selected Rudong County in Eastern China as a case study, choosing coastal tidal flats and croplands with different cultivation durations to compare the effect of reclamation and long-term cultivation on SOC and TN in the coastal plain. The objectives of our study were to (1) analyse the dynamics of SOC and TN following cultivation in the coastal area; (2) indicate the spatial variation of SOC and TN in the coastal area; and (3) calculate carbon sequestration potential in the coastal plain.

## 2. Materials and Methods

### 2.1. Study Area

**Figure 1:**
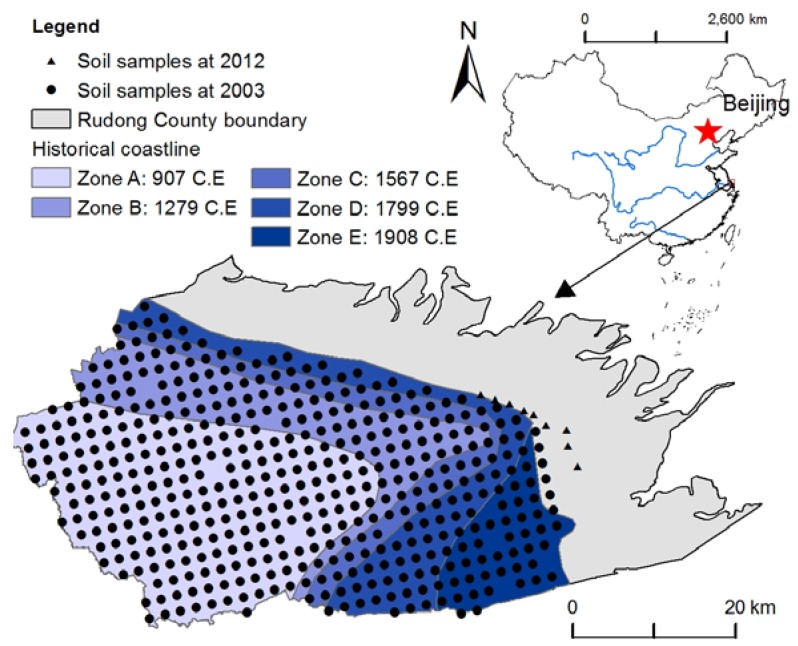
Location of study area and sampling sites.

Rudong County is located next to the Yellow Sea at the mid-northern part of Eastern China (Figure 1). Rudong County was formed by prograding coast from B.C. 220, and this historical coast line is shown in Figure 1 [27]. The coastal tidal flats in Rudong County were dominated by native C_3_ plants, such as *Suaeda salsa*, and invasive species, such as *Spartina alterniflora*. The coastline in Rudong is 106 km long. The tidal flats have an area of 69,300 ha. The climate in the study area is controlled by East Asia monsoons, and the annual mean temperature is 15 °C. The annual mean precipitation is 1028.6 mm. We divided the study area into five zones with different reclamation durations (Figure 1), based on the historical coastal lines.

### 2.2. Soil Collection and Analysis

In 2012, nine samples were collected at a depth of 0–20 cm from a coastal tidal flat covered with *Suaeda salsa*. In 2003, 454 soil samples were collected in the cropland at a depth of 0–20 cm by using a regular grid-sampling scheme with 1000 m spacing (Figure 1).

All samples were air-dried, ground, and passed through 2 mm, 0.25 mm, and 0.149 mm sieves to determine their soil chemical properties. The nine soil samples from outside the embankment were used to analyse the bulk density, organic carbon, total nitrogen, pH, soil particle size, and soil moisture of the coastal tidal flat. The 454 soil samples were used to analyse the organic carbon, total nitrogen, and pH of the cropland. The bulk density of soil samples was measured using a cutting ring method [28]. The SOC was measured using dichromate oxidation [28]. The TN was determined using the Kjeldahl method [28]. Soil pH was determined from soil extracted with distilled water at a 1:5 soil-water ratio using a pH meter (IQ 120, Hach, Loveland, CO, USA). Soil particle size was measured using a laser particle size analyser (Mastersizer 2000, Malvern, Worcs, UK) [29].

### 2.3. Calculation of Soil C and N Density

The SOC density at each sampling site was calculated by using the following equation:

(1) SOCD=BD×SOC

where SOCD is the SOC density, expressed as the SOC per unit area (kg·C·m^−2^), and BD is the soil bulk density.

The following equation was used to calculate soil N density (SND):

(2) SND=BD×SN

where SND is expressed as soil nitrogen per unit area (kg·C·m^−^^2^), and BD is the soil bulk density.

### 2.4. Statistical Analysis

We used the analysis of variation (ANOVA) to test for differences in SOC and TN between different coastal zones with different land uses and reclamation durations. The Pearson correlation analysis was used to indicate the relationships among different soil properties. The ANOVA and Pearson correlation analysis were performed using the software of SPSS 21 (SPSS IBM, Armonk, NY, USA). Geo-statistics were calculated by using the software of ArcGIS 10.0 (ESRI, Armonk, NY, USA). We used ordinary kriging to analyse the spatial distribution of SOC and TN in Rudong County.

## 3. Results

### 3.1. Soil Properties of Uncultivated Coastal Tidal Flat

There was a larger variation in the soil bulk density, soil water, SOC, TN, and contents of sand, silt, and clay in the uncultivated coastal tidal flat (Table 1). The clay content had a larger variation than the content of sand and silt, and ranged from 1.88% to 11.33%. The mean content of silt (50.73%) was larger than that of sand and clay. The sand content ranged from 22.56% to 63.31%. The variation of soil water was larger than those of the bulk density, SOC, TN, and pH. The maximum of the bulk density was very high. The mean of SOC (4.29 g·kg^−1^) was greater than that of TN (0.21 g·kg^−1^). The SOC changed greatly from 1.62 to 3.75 g·kg^−1^. The TN changed only slightly, from 0.15 to 0.25 g·kg^−1^. The soil pH ranged from 8.33 to 9.0, so it was highly alkaline.

There were few significant correlations with soil properties at the uncultivated tidal flat (Table 1). The bulk density was negatively correlated with the clay content and was not correlated with pH, SW, SOC, TN, SAND, and SILT (Table 1). Soil pH had no significant correlation with SOC and TN. SOC was not correlated with TN at the uncultivated coastal tidal flat. Sand content was negatively correlated with silt and clay (*p* < 0.01). Silt content was positively correlated to clay (*p* < 0.05).

### 3.2. SOC and TN Density of Uncultivated Coastal Tidal Flat and Cropland

**Figure 2:**
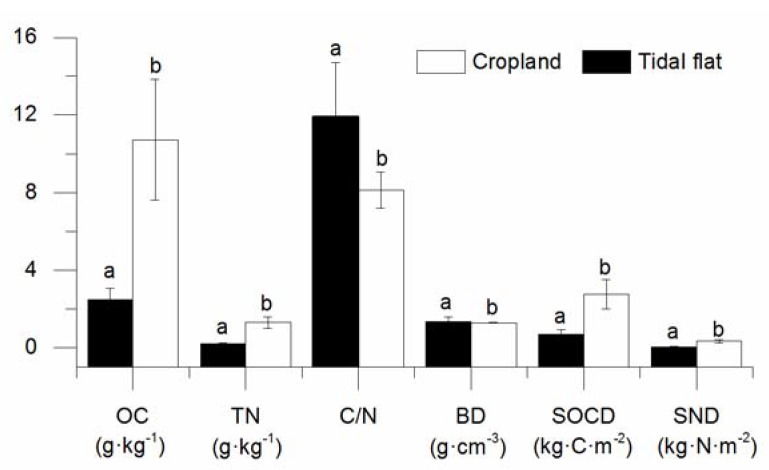
Soil organic carbon (SOC), total nitrogen (TN), C/N, bulk density (BD), soil organic carbon density (SOCD), and soil nitrogen density (SND) of the coastal tidal flat and cropland. Vertical bars denote standard errors of means. Bars with the different letters indicate significant differences at *p* < 0.05.

The SOC and TN at a depth of 0–20 cm were significantly smaller in the uncultivated coastal tidal flat (2.49 g·kg^−1^, 0.21 g·kg^−1^) compared to the cropland (10.72 g·kg^−1^, 1.3 g·kg^−1^) (Figure 2 and Table 2). However, the C/N value at a depth of 0–20 cm in the uncultivated coastal tidal flat was higher (11.17) than that in the cropland (8.14). The rate of increase for the SOC was higher than that of soil total nitrogen from the coastal tidal flat to the cropland. The bulk density of soil from the coastal tidal flat and cropland were significantly different, at 1.36 g·cm^−3^ and 1.29 g·cm^−3^, respectively (Figure 2). The Pearson correlation coefficient of SOC and TN in the cropland was 0.946 (*p* < 0.01) (Table 2). The SOCD of the cropland (2.75 kg·C·m^−^^2^) was higher than that of the coastal tidal flat (0.68 kg·C·m^−^^2^) (*p* < 0.01). The SND of the cropland (0.33 kg·N·m^−^^2^) was higher than that of the coastal tidal flat (0.05 kg·N·m^−^^2^) (*p* < 0.05). 

### 3.3. The Effect of Cultivation Time on SOC and TN of Cropland

Table 3 shows the SOC, TN, and C/N values at different reclamation zones. There were significant differences in SOC, TN, and C/N among the zones of A, B, C, D, and E (*p* < 0.05). The SOC, TN, and C/N values in Zone A with a reclamation duration of 1096 years were 12.98 g·kg^−1^, 1.49 g·kg^−1^, and 8.71, respectively. The SOC, TN, and C/N in Zone E with a reclamation duration of 95 years were 5.76 g·kg^−1^, 0.86 g·kg^−1^, and 6.78, respectively. The SOC, TN, and C/N decreased gradually from Zone A to Zone E. The annual rates of increase for SOC and soil total nitrogen were 0.0073 g·kg^−1^ and 0.0006 g·kg^−1^, respectively.

**Figure 3:**
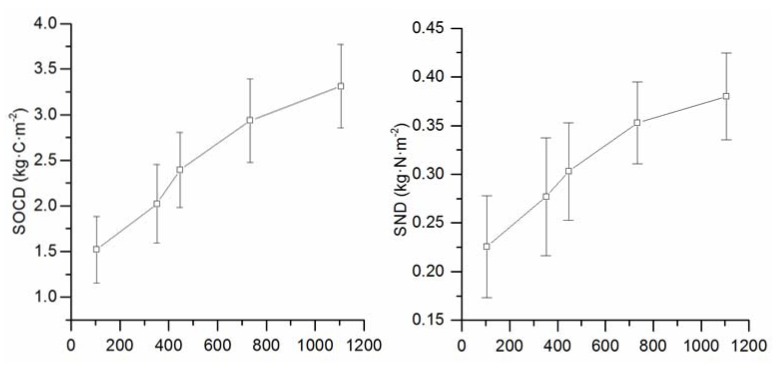
SOCD and SND of cropland with different cultivation duration.

Figure 3 shows that the SOCD and SND of the cropland increased with cultivation duration. The SOCD and SND in Zone E had a cultivation duration of 105 years (1.52 kg·C·m^−^^2^ and 0.23 kg·N·m^−^^2^), which was the lowest among all of the zones. The SOCD and SND in Zone A with a cultivation duration of 1106 years (3.31 kg·C·m^−^^2^ and 0.38 kg·N·m^−^^2^) were the highest among all of the zones. The differences in SOCD and SND between Zones A and E were 1.79 kg·C·m^−^^2^ and 0.15 kg·N·m^−^^2^, respectively.

### 3.4. Mapping Organic Carbon and Total Nitrogen of Soil by Kriging

**Figure 4:**
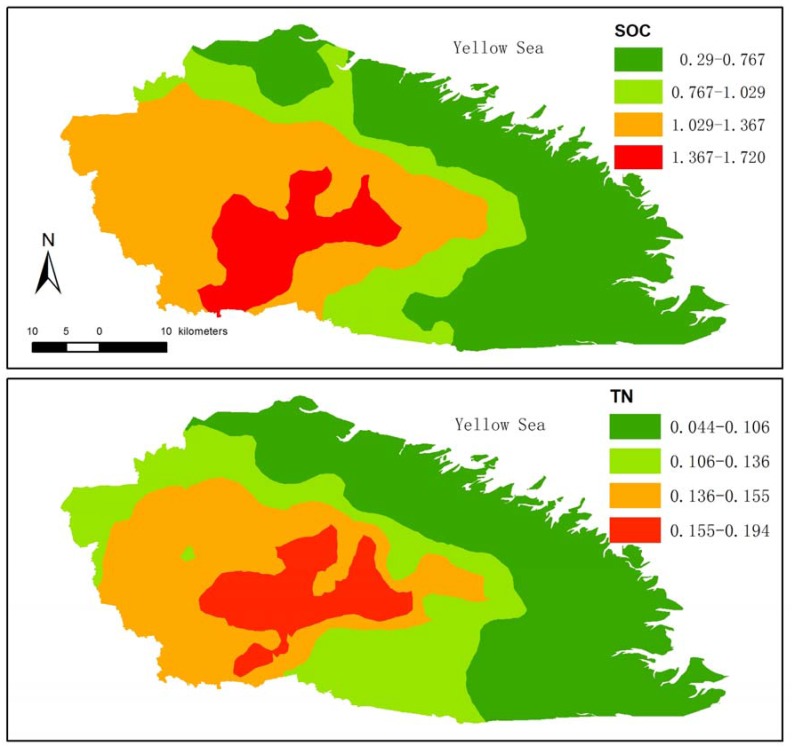
SOC (g·kg^−1^) and TN (g·kg^−1^) mapping of Rudong County.

Table 4 shows the fitted semi-variogram models for SOC and TN. Cross-validation revealed that the spherical model fitted best for SOC and TN. The nugget/sill ratio of organic carbon and total nitrogen was 5% and 10%, respectively, which indicated that SOC and TN had a strong spatial correlation. SOC had a stronger spatial correlation than TN. The range values of SOC and TN were both 0.78 km. Figure 4 shows the mapping of SOC and TN using the kriging method with a spherical model.

## 4. Discussion

### 4.1. OC and TN in Soil of the Coastal Tidal Flat

Soils can sequester CO_2_ to mitigate the greenhouse effect [30]. Soils of coastal tidal flats are scattered around the world and play an important role in carbon sequestration [17]. Globally, the SOC at the coastal tidal flat has a larger variation due to different temperature, vegetation, and soil types [2,17,31]. Soils in mangrove wetlands have a higher capability of carbon sequestration than those in salt marsh wetlands (Table 5). The SOC of bare sediments in the coastal tidal flat was lowest among all of the land types studied (Table 5). The SOC in the coastal tidal flat of our study area was 2.49 g·kg^−1^, which was similar to other studies [9], and lower than that of other coastal wetlands in the Southwestern Pacific and Northwestern Pacific [6,32]. Meanwhile, the correlation of SOC and other soil properties was lower, because of the weak bio-geochemistry process (Table 1). We found that the SOC in the mid-latitude region of the world was lower than those at high and low altitudes (Table 5). Compared to SOC, the TN of coastal tidal flats in our study area was lower. Therefore, the ratio of C/N was very high (Figure 2), which was similar to the results of other studies [28,33,34].

### 4.2. Effect of Land Use Changes on SOC and TN

Land use changes from one ecosystem to another could occur naturally or following human activities [49]. In our study, we found that the SOC and TN increased 145.49% and 519.05%, respectively, after conversion from coastal tidal flats to cropland (Figure 2). Soils in the cultivated land and coastal tidal flats had different redox conditions, which could lead to organic carbon and nitrogen mineralization and decomposition under aerobic conditions [6]. Many scholars have also found that SOC initially declined after reclamation and then increased to high levels [40,50], and this was consistent with our study. However, our results contradicted those of other studies by showing that cultivation promotes SOC loss by exposing micro-aggregate SOC to microbial decomposition and by changing the moisture and temperature [51,52]. The SOC declines slightly from coastal wetland to cropland (−6.9%) [6] and decreases greatly from forest to cropland (−42%) and from pasture to cropland (−59%) [49]. The results of our study agreed with those of other studies by showing that soils with very low carbon content tended to gain slight amounts of carbon after cultivation, but that soils with high carbon lost at least 20% of their carbon during cultivation [53]. The increase in SOC and TN from the coastal tidal flats to the cultivated land was due to the relatively low carbon and nitrogen of the initial natural coastal wetland that was covered with scarce *Suaeda salsa*, as well as farmers’ preserved crop residues in the cropland, which increased soil biomass. We also found that the C/N value in the coastal tidal flats (11.17) was higher than that in the cropland (8.14) due to the lower rate of increase for SOC and the higher rate of increase for nitrogen. This finding highlights the importance of the initial soil conditions on the conversion from coastal tidal flats to other land use types and the long-term effect of land use change on soil carbon sequestration.

### 4.3. SOC and TN Following Long-Term Cultivation

Carbon sequestration in agricultural soil was accountable under the Kyoto Protocol [54]. Many scholars have identified and quantified the effects of human activities on soil carbon [11,38,54]. Soil disturbance by tillage was a primary cause of SOC loss [11,55]. However, conservation tillage with less intensive plowing only led to a higher concentration near the surface, whereas conventional tillage sequestrated carbon in deeper layers [56]. Some studies indicated that high residue input from fall and winter crops is important for adding organic carbon and nitrogen to the soil [50]. In our study area, the SOC and TN were higher in old cropland than young cropland due to the effects of cultivation over a long time scale, indicating an accumulation of SOC and nitrogen by cultivation (Table 2). The rate of increase for SOC and TN sequestration with cultivation time in our study was 0.0073 g·C·kg^−1^·year^−1^ and 0.0006 g·N·kg^−1^·year^−1^, respectively. The increase rates of organic carbon and nitrogen in our study were less than results from other studies, which ranged from 0.13–0.22 g·C·kg^−1^·year^−1^ and 0.007–0.013 g·C·kg^−1^·year^−1^, respectively [28,40], due to the different time scales. The annual rate of increase for organic carbon and nitrogen over the long term was less than that over the short term. The spatial distribution of SOC was consistent with that of historic coastal line (Figure 4), which indicated that the space for time substitution was feasible for analysing the dynamics of SOC. In our study, we found that the cropland cultivated by conventional tillage management and crop systems was a carbon and nitrogen sink. The C/N value increased after cultivation due to the rate of increase for SOC being higher than that for soil nitrogen.

### 4.4. The Potential of Soil Carbon Sequestration at the Reclaimed Coastal Tidal Flats

The SOC density of the reclaimed coastal tidal flats in our study area was lower than that of other coastal tidal flats [17]. Meanwhile, the SOC density of the cropland at the reclaimed coastal tidal flat in our study area was lower than both that of average cropland soils [57] and the regional average [58]. The lower SOC density in our study area indicated high soil carbon sequestration potential [28,59]. A significant increase in SOCD and SND was found in the top 20 cm of soil in the cropland compared to the coastal tidal flats and in the older cropland compared to the young cropland (Figure 2). The changes in SOCD and SND were highly dependent on management practices, such as the reclamation history, tillage management and application of soil amendments. In contrast, the significant differences in SOCD and SND among coastal tidal flats, young cropland and old cropland over a longer time scale indicated increasing SOCD and SND after cultivation. With the development of soil properties after reclamation and cultivation, the SOC density in the coastal tidal flats and the young cropland with land management will recover to those of the old cropland. Within the same climate zone, the high SOC density of the old cropland can provide a reference for carbon sequestration capacity. Therefore, by using the maximum SOC contents of five reclamation zones as the organic carbon saturation points, the maximum SOC density potential at the five reclamation zones was 2.68 kg·C·m^−^^2^.

## 5. Conclusions

The SOC and TN of coastal tidal flats at the mid-latitude were lower than that at the high and low altitude. Reclamation of coastal tidal flats to cropland increased both the SOC and TN, which is beneficial for mitigating the greenhouse effect and improving food security. Cultivation improved the potential for soil carbon sequestration in the reclaimed coastal tidal flat. In the future, coastal management should consider the spatial distribution of SOC and TN. By using different cultivation management techniques, such as tillage, crop systems will be required to improve soil properties and enhance the potential capability of soil carbon sequestration in different regions.

## Figures and Tables

**Table 1:** Pearson correlation and classical statistics of soil properties of the samples (*n* = 9) from the uncultivated coastal tidal flat.

	BD	pH	SW	SOC	TN	Sand	Silt	Clay
BD	1							
pH	0.159	1						
SW	0.119	0.541	1					
SOC	−0.004	0.161	0.145	1				
TN	−0.567	0.379	−0.012	0.197	1			
Sand	0.578	0.262	0.416	−0.081	−0.298	1		
Silt	−0.517	−0.255	−0.359	0.057	0.271	−0.991 **	1	
Clay	−0.717 *	−0.237	−0.570	0.166	0.353	−0.830 **	0.749 *	1
Minimum	0.94	8.33	9.21	1.62	0.15	22.56	34.81	1.88
Maximum	1.66	9.00	20.63	3.75	0.25	63.31	66.12	11.33
Mean	1.36	8.67	13.51	2.49	0.21	44.29	50.73	4.97
SE	0.08	0.09	1.16	0.59	0.00	4.53	3.81	0.91
CV	0.17	0.03	0.26	0.23	0.15	0.31	0.23	0.55
Skewness	−0.67	0.05	1.09	0.94	−0.68	0.06	−0.31	1.73
Kurtosis	−0.10	−1.59	1.21	2.59	0.06	−0.85	−1.46	3.96

Note: BD: bulk density (g·cm^−3^); SW: soil water (%); SOC: organic carbon (g·kg^−1^); TN: total nitrogen (g·kg^−1^). The soil particle sizes of clay, silt, and sand are <0.005 mm, 0.005–0.05 mm, and >0.05 mm, respectively (U.S. Bureau of Soils); *: correlation is significant at 0.05 (2-tailed); **: correlation is significant at 0.01 (two-tailed); SE: standard error; CV: coefficient of variation.

**Table 2:** Pearson correlation and classical statistics of SOC and TN of the samples (*n* = 454) from the cropland

	OC	TN	Minimum	Maximum	Mean	SE	CV	Skewness	Kurtosis
OC	1.000		2.90	17.20	10.72	3.12	0.29	−0.28	−0.70
TN	0.946 **	1.000	0.44	1.94	1.30	0.29	0.23	−0.54	−0.02

**: correlation is significant at the 0.01 level (two-tailed).

**Table 3:** SOC, TN, and C/N ratio of 454 soil samples.

	SOC (g·kg^−1^)		TN (g·kg^−1^)		C/N	
	Mean	Std	Mean	Std	Mean	Std
Zone E (*n* = 48)	5.76a	1.43	0.86a	0.20	6.78a	0.79
Zone D (*n* = 70)	7.74b	1.70	1.06b	0.24	7.38b	0.88
Zone C (*n* = 45)	9.22c	1.64	1.17c	0.20	7.91c	0.52
Zone B (*n* = 92)	11.42d	1.87	1.37d	0.17	8.30d	0.60
Zone A (*n* = 199)	12.98e	1.89	1.49e	0.19	8.71e	0.58

Note: values with different letters (a, b, c, d, e) indicate significant differences at *p* < 0.05. The Std is the abbreviation of standard deviation.

**Table 4:** Parameters of different Kriging models of organic carbon and nitrogen.

	Model	Nugget	Sill	Nugget/Sill	Range	Cross Validation			
						MS	RMS	ASE	RMSS
SOC	circular	0.01	0.27	0.05	0.78	0.00	0.15	0.16	0.98
	spherical	0.01	0.23	0.05	0.78	0.00	0.15	0.16	1.00
	Exponential	0.00	0.19	0.00	0.78	0.00	0.15	0.12	1.37
	Gaussian	0.04	0.27	0.13	0.69	0.00	0.16	0.22	0.77
N	circular	0.01	0.15	0.09	0.78	0.01	0.02	0.02	0.91
	spherical	0.01	0.13	0.10	0.78	0.00	0.02	0.02	0.92
	Exponential	0.01	0.11	0.05	0.78	−0.01	0.02	0.02	1.06
	Gaussian	0.03	0.17	0.17	0.78	0.03	0.02	0.02	0.78

Note: MS, RMS, ASE, and RMSS are the abbreviation of Mean Square, Root Mean Square, Average Standardized Error, and Root Mean Square Standardized.

**Table 5:** SOC of salt marshes at different coastal tidal flats in the world.

		Index	Depth	Value	Date Source
Gulf of Mexico	Salt marsh	CD	0–2 cm	0.010–0.190	[17]
	Mangrove	CD	0–2 cm	0.024–0.071	[17]
	Coastal wetlands	OC	0–15 cm	2.03–34.69	[34]
	Freshwater marsh	OC	0–20 cm	137.3	[35]
	Freshwater swamp	OC	0–20 cm	200	[35]
Pacific and India Ocean	Mangrove	CD	0–2 cm	0.023–0.040	[17]
	Native forest	OC	0–10 cm	60.8	[36]
	Perennial pasture	OC	0–10 cm	30.1	[36]
Northeast Atlantic	Salt marsh	CD	0–2 cm	0.020–0.041	[17]
Southwest Atlantic	Salt marsh	OC	0–5 cm	67	[37]
	Bare sediment	OC	0–5 cm	14	[37]
Northwest Atlantic	Salt marsh	CD	0–2 cm	0.018–0.078	[17]
Mediterranean	Salt marsh	CD	0–2 cm	0.073	[17]
Northeastern Pacific	Salt marsh	CD	0–2 cm	0.009–0.040	[17]
Southeastern Pacific	Cropland	TC	0–2.5 cm	4.6–7.4	[38]
	Cropland	OC	0–5 cm	4.96	[39]
Southwestern Pacific	Rice paddy	OC	0–7 cm	17.8	[22]
	Cropland	OC	0–20 cm	13.48	[6]
	Salt marsh	OC	0–13 cm	10.9	[22]
	Salt marsh	OC	0–20 cm	15.44–21.83	[6]
	Bare sediment	OC	0–30 cm	5.1	[22]
Mid-western Pacific	Salt marsh	OC	0–8 cm	15.68	[40]
	Salt marsh	OC	0–20 cm	1.28	[41]
	Salt marsh	OC	0–30 cm	1–10	[42]
	Tidal flat	OM	0–20 cm	4.78	[43]
	Salt marsh	OC	5–20 cm	4–8.23	[44]
	Bare sediment	OC	0–20 cm	0.69	[45]
	Tidal flat	OC	0–20 cm	2.49	our study
	Cropland	OC	0–20 cm	5.76–12.98	our study
	Cropland	OC	0–8 cm	5.70–24.35	[40]
	Cropland	OC	0–20 cm	4.2–22.5	[31,46]
Northwestern Pacific	Salt marsh	OM	0–20 cm	130.09	[32]
	Salt marsh	OC	0–15 cm	9.09	[47]
	Salt marsh	OC	0–60 cm	2.15–5.00	[48]
	Bare sediment	OC	0–15 cm	6.68	[47]
	Rice paddy	OM	0–20 cm	29	[32]

Note: CD, density of carbon (g·cm^−3^); OC, organic carbon (g·kg^−1^); OM, organic matter (g·kg^−1^).
